# Mass Spectrometry-Based Strategies for Assessing Human Exposure Using Hemoglobin Adductomics

**DOI:** 10.1021/acs.chemrestox.3c00294

**Published:** 2023-11-14

**Authors:** Andrew T. Rajczewski, Lorena Ndreu, Efstathios Vryonidis, Alexander K. Hurben, Sara Jamshidi, Timothy J. Griffin, Margareta Å. Törnqvist, Natalia Y. Tretyakova, Isabella Karlsson

**Affiliations:** † Department of Biochemistry, University of Minnesota, Minneapolis, Minnesota55455, United States; ‡ Department of Environmental Science, Stockholm University, SE-10691Stockholm, Sweden; § Department of Medicinal Chemistry and the Masonic Cancer Center, University of Minnesota, Minneapolis, Minnesota55455, United States

## Abstract

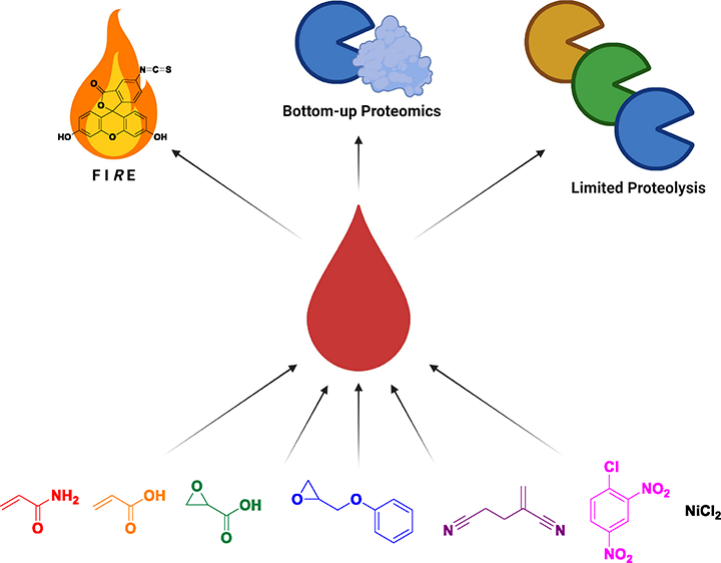

Hemoglobin (Hb) adducts are widely used in human biomonitoring due to the high abundance of hemoglobin in human blood, its reactivity toward electrophiles, and adducted protein stability for up to 120 days. In the present paper, we compared three methods of analysis of hemoglobin adducts: mass spectrometry of derivatized N-terminal Val adducts, mass spectrometry of N-terminal adducted hemoglobin peptides, and limited proteolysis mass spectrometry . Blood from human donors was incubated with a selection of contact allergens and other electrophiles, after which hemoglobin was isolated and subjected to three analysis methods. We found that the FI*R*E method was able to detect and reliably quantify N-terminal adducts of acrylamide, acrylic acid, glycidic acid, and 2,3-epoxypropyl phenyl ether (PGE), but it was less efficient for 2-methyleneglutaronitrile (2-MGN) and failed to detect 1-chloro-2,4-dinitrobenzene (DNCB). By contrast, bottom-up proteomics was able to determine the presence of adducts from all six electrophiles at both the N-terminus and reactive hemoglobin side chains. Limited proteolysis mass spectrometry, studied for four contact allergens (three electrophiles and a metal salt), was able to determine the presence of covalent hemoglobin adducts with one of the three electrophiles (DNCB) and coordination complexation with the nickel salt. Together, these approaches represent complementary tools in the study of the hemoglobin adductome.

## Introduction

Protein adductomics represents an invaluable resource in the fields of exposomics and toxicology. In contrast to DNA adducts that can be rapidly removed via several base excision repair processes,^1^ and barring those reversible electrophiles that serve signaling/inhibitory functions^2,3^ or are chemically unstable,^4^ many exogenous electrophiles will react with a protein and remain as adducts until the recycling of the protein via proteasomal degradation.^5^ Because of the relative longevity of many proteins, these protein adducts can accumulate in the body over longer periods of time than DNA adducts, making them ideal reservoirs for studying exposure levels in human and animal subjects.^6,7^ Of the large number of proteins with potential use in adductomics, hemoglobin (Hb) represents a strong candidate due to its long lifetime and its high abundance in the blood.^8^ Several alternative methods exist for profiling the hemoglobin adductome in response to exposure to organic chemicals, including the detection of N-terminal Val adducts and the analysis of modified peptides following tryptic digestion. Additionally, there is a need to detect protein adducts with metal ions as they can influence protein structure and elicit immune responses in patients.^9^ The goal of this investigation was to compare the available methods for surveying hemoglobin adducts by mass spectrometry-based methods.

Pioneered by the Törnqvist lab^10^ at Stockholm University, GC/MS or LC/MS analysis of adducts (R) to N-terminal valines in hemoglobin following modified Edman degradation has shown considerable utility for screening populations for exposure to electrophiles.^11,12^ Of these methods, the FI*R*E method (using fluorescein isothiocyanate for the detachment of N-terminal adducts and LC/MS analysis) shows great promise to detect and quantify N-terminal adducts within hemoglobin. However, this method is able to detect the formation of adducts only at the N-terminal Val of hemoglobin. Furthermore, it is not known whether all electrophiles can be detected with equal efficiency using FI*R*E, as it is known that bulky and bifunctional modifications of N-terminals can be resistant to the Edman degradation in peptide sequencing.^13^ It has also been shown that the ring-closed N-terminal valine adduct of the bifunctional metabolite diepoxybutane cannot be used to monitor butadiene exposure using the modified Edman degradation.^14,15^ The Tretyakova laboratory previously employed bottom-up proteomics to detect 4-hydroxybenzyl adducts in hemoglobin.^16^ This earlier study^16^ demonstrated that bottom-up proteomics could be used as an alternative method to detect the adducts at the N-termini of hemoglobin. We observed the N-terminal Val adducts previously detected by the FI*R*E method but also showed adduct formation at several nucleophilic side chains in hemoglobin, illustrating its capacity as adduct reservoir, which can be explored for screening of exposure.^16^ The availability of several nucleophilic sites within hemoglobin for adduct formation is also illustrated by results from other research groups that have started exploring proteomics for studies of adducts to hemoglobin, particularly from endogenous reactions, such as oxidation and nitration. For example, Kojima et al. developed peptide-based methods to detect oxidized, nitrated, lipidated, and glycated sites within human globin,^17^ while Chen et al. observed acrolein-induced modifications in hemoglobin of smokers and nonsmokers.^18^

Bottom-up proteomics and FI*R*E can detect covalent adducts in hemoglobin; however, for metal salts, there is the possibility of coordination complexation with proteins, which could be disrupted during the FI*R*E procedure or during tryptic digestion. The formation of covalent modifications^19,20^ and noncovalent interactions^21^ alike results in changes to the protein conformation and can induce immune response.^9,22^ Noncovalent and coordination complexes of hemoglobin with small molecules and metal ions can be detected through limited proteolysis mass spectrometry (LiP-MS), in which samples are briefly digested with proteinase K prior to being digested with trypsin.^23^ By comparing the results of these digests with a control treated with solvent only, sections of hemoglobin with altered solvent exposure due to the presence of an adduct on the protein can be ascertained.

**Figure 1:**
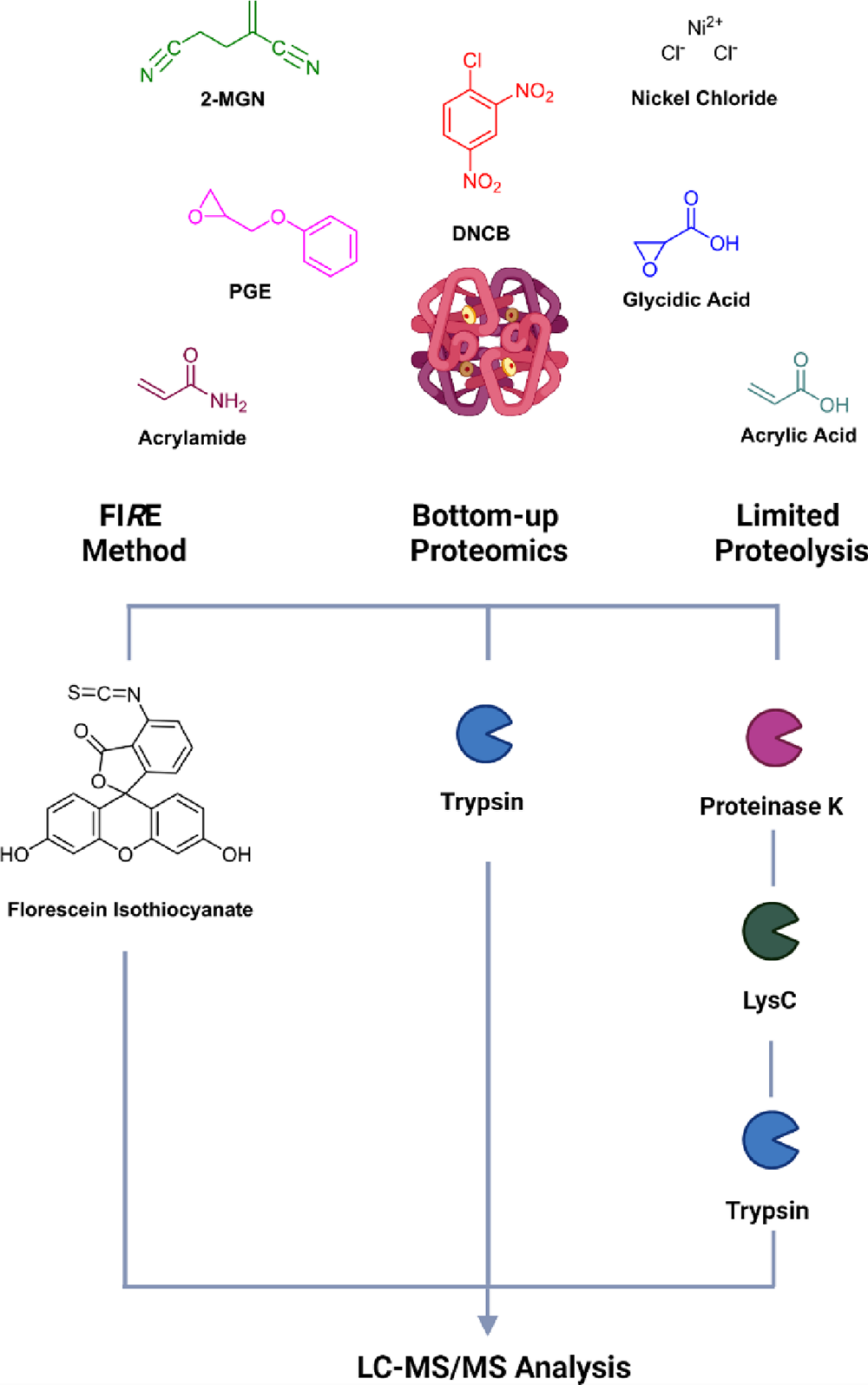
Strategies for examining the adduct formation in hemoglobin. In the FI*R*E method, N-terminal hemoglobin adducts are derivatized and separated from the rest of the molecule before LC-MS analyses. In bottom-up proteomics, hemoglobin is digested enzymatically using trypsin before LC-MS and bioinformatic analysis. In contrast to bottom-up proteomics, limited proteolysis mass spectrometry relies on the use of multiple proteases and comparison against a known standard to demonstrate exposure-driven changes in protein conformation.

In the present study, we investigated and compared the performance of three strategies, the FI*R*E method, bottom-up proteomics, and LiP-MS in characterizing hemoglobin adducts with various electrophiles and one metal salt (Figure 1). In this work, we compared the ability of these methods to detect adducts formed at hemoglobin nucleophilic sites following the exposure of blood to electrophiles at varying concentrations and incubation times as well as assess hemoglobin structural changes after incubation with compounds not forming stable covalent adducts. Ultimately, we were able to determine the relative utility of each method and how each would be best used in querying the hemoglobin adductome.

## Materials and Methods

### Materials

For initial electrophile exposures, blood was acquired from the Karolinska Universitets Laboratoriet in Stockholm. Acrylamide, acrylic acid, potassium oxirane-2-carboxylate, 1-chloro-2,4-dinitrobenzene, 2,3-epoxypropyl phenyl ether, 2-methyleneglutaronitrile, triethylammonium bicarbonate, ammonium bicarbonate, nickel chloride, and deoxycholic acid were purchased from Millipore Sigma (Burlington, MA). BCA Assay kit, Pierce C18 spin columns, dimethylformamide, iodoacetamide, and dithiothreitol were purchased from Thermo Fisher Scientific (Waltham, MA). Additional C18 desalting columns were purchased from G-Biosciences (St. Louis, MO). Proteinase K was purchased from New England Biolabs (Ipswitch, MA). Oasis MAX 3 cc SPE cartridges and Sep-Pak C18 spin columns were obtained from Waters (Milford, MA). Bead-immobilized and solvated Trypsin and Endoproteinase Lys-C were purchased from Promega Corporation (Madison, WI). Fluorescein isothiocyanate (FITC) isomer I (CAS: 3326–32–7) was obtained via Chemtronica, Sweden as well as Millipore Sigma (Burlington, MA).

### Exposure of whole blood to electrophiles

Aliquots of donor blood (500 μL) were incubated with individual electrophiles using different molar ratios and incubation times, as documented in Table 1. All incubations were conducted at 750 rpm at 37 °C.

**Table 1:**
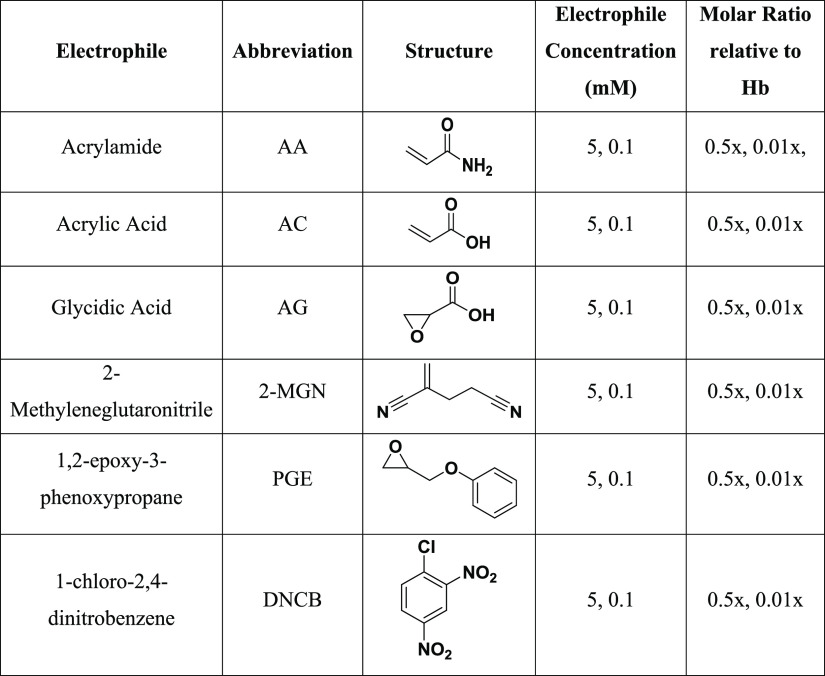
Incubation Conditions of Whole Blood for FIRE and Bottom-up Proteomics Analysis^a^

a All incubations were conducted for 1 and 21 h.

### Isolation of erythrocytes

Following incubation, hemoglobin-containing fraction was isolated from fresh human blood incubated with electrophiles or nontreated control samples, according to published protocols.^24^ Briefly, 0.5 mL aliquots of incubated human blood were centrifuged at 800*g* for 15 min at 4 °C to separate the erythrocytes from the plasma. The plasma was decanted from the pelleted erythrocytes, which were then subjected to three washes with an equal volume of cold 0.1% NaCl_(aq)_ followed by further centrifugation. Erythrocytes were lysed by suspension in an equal volume of distilled water and subjected to 5 min of sonication. Samples were then centrifuged at 11,000 rpm for 10 min to pellet cellular debris, reserving the hemoglobin-containing supernatant for further experiments. Hemoglobin concentration was ascertained via the HemoCue Hb 201+ system.

### FIRE Method Protocol

The samples were derivatized according to the FI*R*E procedure^10^ with slight modifications. Following the lysis of the erythrocytes, samples of control or exposed hemoglobin were added to 2.0 mL Eppendorf tubes to a final volume of 250 μL and a final concentration of 100–150 g/L. To these were added 15 μL of fresh 1 M KHCO_3_ and 30 μL of FITC stock solution (5 mg solved in 30 μL DMF). Samples were then incubated overnight at 37 °C with 750 rpm. Following overnight incubation, 2 pmol of D_7_-labeled internal standard (AA-Val(D_7_)-FTH) were spiked into each sample after which the remaining protein was precipitated via the addition of 1.5 mL of acetonitrile followed by vortexing and centrifugation at 3000 g for 10 min. The sample supernatants were made basic with the addition of 25 μL of 1 M NH_4_OH and loaded on Oasis MAX 3 cc SPE cartridges that were preconditioned with 0.01 M NH_4_OH. Samples were then washed with 1 column volume of acetonitrile, 1 column volume of water, and 0.5 column volume of 0.5% cyanoacetic acid in water, after which air was blown through each cartridge to remove excess solvent. FTH-analytes were eluted with 1.1 mL of 0.25% cyanoacetic acid in 60% acetonitrile, after which the samples were dried overnight under nitrogen stream. Prior analysis with LC-MS, the samples were reconstituted in 100 μL 40% ACN in water.

### Digestion and Processing for Bottom-up Proteomics

Following incubation, aliquots of protein (50 μg) were taken and buffer-exchanged three times in TEAB buffer. Buffer-exchanged hemoglobin samples were treated with 10-fold molar excess iodoacetamide in TEAB buffer in the dark at room temperature for 30 min. Each sample was then supplemented with trypsin at a ratio of 1:20 w/w and incubated overnight at 37 °C. Proteolytic digestion was terminated via the addition of formic acid to 10%, after which the samples were desalted via Pierce C18 spin columns and evaporated to dryness under vacuum.

### Incubation of Electrophiles for Limited Proteolysis and Limited Proteolysis Protocol

Limited proteolysis mass spectrometry experiments were conducted on isolated hemoglobin following previously reported methodologies.^23^ Briefly, hemoglobin from lysed erythrocytes was aliquoted in phosphate-buffered saline and incubated with 5-fold molar excess of DNCB, PGE, 2-MGN, NiCl_2_, or a DMSO control for 4 h at 37 °C. Following incubation, the samples were processed with Zeba spin desalting columns per the manufacturer’s instructions to remove excess small molecules. Next, 100 μg aliquots of each sample were incubated with 1 μg of proteinase K at 25 °C for 1 min, and then were transferred to a 95 °C water bath for 5 min to inactivate the proteinase K. Following proteinase K incubation, deoxycholic acid was added to a final concentration of 5% (wt/vol). Dithiothreitol was then added to a final concentration of 12 mM and the samples were incubated at 30 °C for 30 min. Iodoacetamide was then added to 40 mM, after which samples were incubated in darkness at room temperature for 45 min. The samples were then digested with LysC (1 μg) for 4 h at 37 °C and then diluted to 500 μL with 100 mM NH_4_HCO_3_ to which trypsin was added (1 μg). The samples were incubated overnight at 37 °C. Following digestion, deoxycholic acid was precipitated through the addition of 2% formic acid. The samples were centrifuged at 16000*g* for 10 min at room temperature and the supernatant was carefully removed. Next, supernatants were desalted with Sep-Pak C18 spin columns per the manufacturer’s guidelines. The samples were then dried under a nitrogen stream prior to MS analysis.

### LC-MS Conditions

#### LC-MS Conditions for the FI*R*E Procedure

For the samples subjected to the FI*R*E procedure, two LC-MS analyses were performed. To determine the exact mass of the precursor and product ions for each analyte, samples were run on a QExactive Orbitrap Hybrid Mass Spectrometer interfaced with a Dionex UltiMate 3000 UHPLC and plumbed with a ACE Excel C18-PFP column (75 × 2.1 mm internal diameter, particle size 1.7 μm, from Advanced Chromatography Technologies Ltd. (Aberdeen, Scotland)). The mobile phases consisted of 90% water with 10% acetonitrile and 0.1% formic acid (solvent A) and 90% acetonitrile with 10% water and 0.1% formic acid (solvent B) and separation was performed at a flow rate of 0.3 mL/min. Samples were run in positive ion mode by using the Parallel Reaction Monitoring (PRM) scan mode. The expected chromatographic peak width was 15 s. PRM experiments were performed at a resolution of 30000, an AGC target of 2 × 105, maximum IT of 100 ms, isolation window of 2 *m*/*z*, and normalized collision energy of 30. Once the appropriate transitions were determined, targeted detection of Val-FTH analytes in FI*R*E samples was performed using a Waters Xevo TQ-S mass spectrometer interfaced an Acquity UPLC System plumbed with an ACE Excel C18-PFP column (75 × 2.1 mm internal diameter, particle size 1.7 μm). The mobile phases used were 95% water with 5% acetonitrile and 0.1% formic acid (mobile phase A) and 95% acetonitrile with 5% water and 0.1% formic acid (mobile phase B). A gradient of was applied beginning at 20% B and increasing to 100% B over 25 min, with 5 min of 100% B before re-equilibration at 20% B for 5 min. Transitions involving the electrophile moiety were monitored for the deuterated IS, AA-Val(D_7_)-FTH (567.2 *m*/*z* to 496.2 *m*/*z*) as well as AA-Val-FTH (560.1 *m*/*z* to 489.2 *m*/*z*), AC-Val-FTH (561.1 *m*/*z* to 489.2 *m*/*z*), AG-Val-FTH (577.1 *m*/*z* to 489.2 *m*/*z*), PGE-Val-FTH (639.2 *m*/*z* to 489.2 *m*/*z*), 2-MGN-Val-FTH (595.1 *m*/*z* to 489.2 *m*/*z*), and DNCB-Val-FTH (655.1 *m*/*z* to 489.2 *m*/*z*) for a total of seven transitions.

#### LC-MS Conditions for Bottom-up Proteomics

Bottom-up proteomics nanoLC-NSI-MS/MS analyses were performed on a QExactive Orbitrap Hybrid Mass Spectrometer interfaced with an Ultimate 3000 UHPLC run in nanoflow mode at 300 nL/min. For separations, the nanoLC column was packed with Luna 5 μm C18 stationary phase, with mobile phases of 0.1% formic acid in water (mobile phase A) and 0.1% formic acid in acetonitrile (mobile phase B). For global proteomics, samples were analyzed using a gradient of 5–22% buffer B over 71 min, followed by 22–33% over 5 min, 33–90% over 5 min, a 90% buffer B wash for 4 min, and finally a 90–4% decrease in buffer B over 2 min followed by a 4 min equilibration at 4% B. Global proteomics experiments were run in the positive ion mode using Full MS/dd-MS^2^ and top 15 mode, 70,000 MS^1^ resolution with an AGC target of 1 × 10^6^, maximum IT of 30 ms, and scan range of 300 to 2000 *m*/*z*. Tandem mass (MS^2^) spectra were captured at 17,500 resolution, AGC target of 5 × 10^4^, maximum IT of 50 ms, isolation window of 2.0 *m*/*z*, and normalized collision energy of 30%. Data were collected in the centroid mode. Control hemoglobin samples were used to create an exclusion list of unmodified hemoglobin peptides. For targeted proteomics experiments, inclusion lists were generated based on manually annotated and validated peptides in the global proteomics experiments. Targeted experiments were conducted in PRM mode with 70,000 MS^1^ resolution, an AGC target of 2 × 10^5^, maximum IT of 250 ms, aisolation window of 1.0 *m*/*z*, and normalized collision energy of 25%.

#### LC-MS Conditions for Limited Proteolysis MS

Limited proteolysis mass spectrometry was conducted on a QExactive Orbitrap Hybrid Mass Spectrometer interfaced with a Dionex UltiMate 3000 UHPLC system plumbed with an Acclaim RSLC 120 C18 (2.2 μm, 120 Å, 2.1 mm × 150 mm, Thermo Scientific Sunnyvale, CA, USA) column. Peptides were separated at a flow rate of 0.3 mL/min on a 45 min gradient with 5–50% buffer B (0.1% FA in acetonitrile) over 30 min followed by 50–95% over 5 min, a 95% buffer B wash for 5 min, and finally a 95–5% decrease in buffer B followed by a 5 min equilibration. Peptides were analyzed in positive ion mode using a Top12 Full MS/dd-MS^2^ experiment with an expected chromatographic peak fwhm of 15 s. In the full MS, resolution was set to 70,000 with an AGC target of 1 × 10^6^, a maximum IT of 30 ms, and a scan range of 300 to 2000 *m*/*z*. Tandem mass spectra were captured at 17,500 resolution, AGC target of 5e4, maximum IT of 50 ms, an isolation window of 2.0 *m*/*z*, and a normalized collision energy of 30%. Data were collected in centroid mode.

### Data Analysis

Once the FI*R*E samples were analyzed via LC-MS, the data were manually interrogated using the QualBrowser software suite from Xcalibur (Thermo Fisher Scientific). Bottom-up proteomics data were analyzed using Proteome Discoverer v2.2 (Thermo Fisher Scientific) where they were searched against the Uniprot FASTA sequences for human hemoglobin α chain and human hemoglobin β chain with the N-terminal methionine residues removed. Acrylamide, acrylic acid, glycidic acid, PGE, 2-MGN, and DNCB were set as variable modifications that could occur at protein N-termini and side chains of cysteine, methionine, histidine, aspartate, arginine, lysine, tyrosine, serine, and threonine. Carbamidomethylation of cysteine was also set as a variable modification. Targeted proteomics data were interrogated using the QualBrowser software suite. For LiP-MS data, MaxQuant^25^ v1.6.5 was used in data processing. Default parameters were applied, and human hemoglobin FASTA sequences from Uniprot were searched against. Variable modifications of methionine oxidation, cysteine carbamidomethylation, and adducts of DNCB, 2-MGN, and PGE were included. To obtain label-free peptide quantification, default LFQ parameters were selected with normalized peptide intensities as the output. The Perseus software suite^26^ (version 1.6.6.0) was used to process the resultant LFQ intensities. The data were Log2 transformed and filtered following previously described methods.^27^ A two-tailed, two-sample *t* test was performed to compare peptide abundance between the electrophile treatment groups and the DMSO control. Statistically significant enrichment was determined with a Benjamini–Hochberg corrected FDR of 0.05 and a minimal coefficient of variation (S0) of 0.1.

## Results

### FI*R*E Method for Detection of the Formation of Adducts at the N-Termini of Hemoglobin

**Figure 2:**
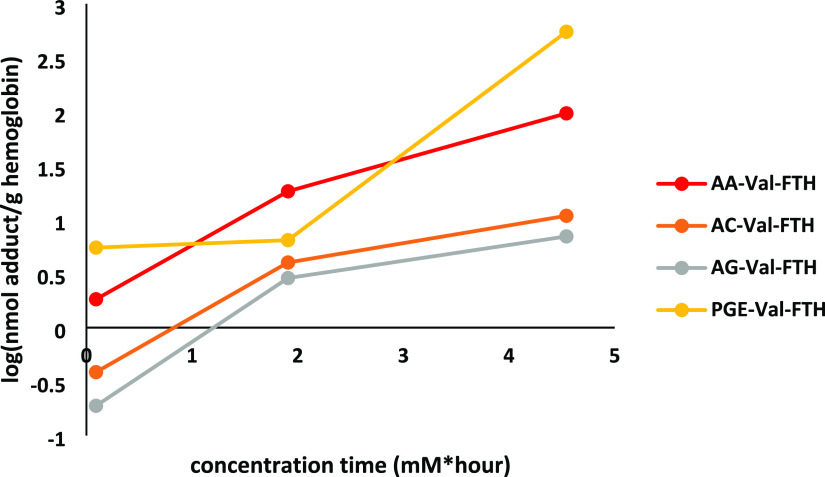
Detection of the N-terminal Valine Hb adduct formation via the FI*R*E method. Semiquantitative dose–response curves of blood samples incubated with electrophiles and processed via the FI*R*E method prior to LC-MS analysis.

Following the exposure of blood samples to the six electrophiles and the isolation of hemoglobin, samples were subjected to the FI*R*E method to determine the ability of FI*R*E to detect assorted kinds of potential electrophiles. Following LC-MS analysis, Val-FTH derivative signals were normalized to the 2 pmol of spiked-in AA-Val(D_7_)-FTH and plotted as a function of concentration x time (expressed as mM*hour) (Figure 2).

Using the FI*R*E method, we were able to see adduct formation at the N-termini of hemoglobin of most of the electrophiles incubated with blood (Figure 2). The adduct levels were quantified by comparing with an IS for the acrylamide adduct, and the adduct levels were roughly estimated to range from ca. 100 pmol/g hemoglobin (from 5 mM 2-MGN incubation for 1 h) to ca. 500 nmol/g hemoglobin (5 mM PGE incubation for 1 h). High doses were used to ascertain the detection of adducts with both analytical methods used. Levels of the adducts from the other electrophiles are more semiquantitative. Four of these six electrophiles were observed to have positive dose–response relationships, with increasing adduct formation with an increasing concentration of electrophiles over time. Of these, PGE and acrylamide were observed to be the most reactive due to the highest levels of adduct formation observed, with acrylic acid and glycidic acid showing lower levels of reactivity. While 2-MGN was observed to form adducts with incubation in blood, it formed far lower levels of adducts relative to the electrophiles as detailed in Figure 2 andFigure S1a, potentially due to its large size impeding adduct formation (Figure S2b). Importantly, DNCB was not observed to form adducts with N-termini in hemoglobin at any concentration and length of incubation time (Figure S1b).

### Bottom-up Proteomics Can Detect Adducts from All Six Electrophiles

**Figure 3:**
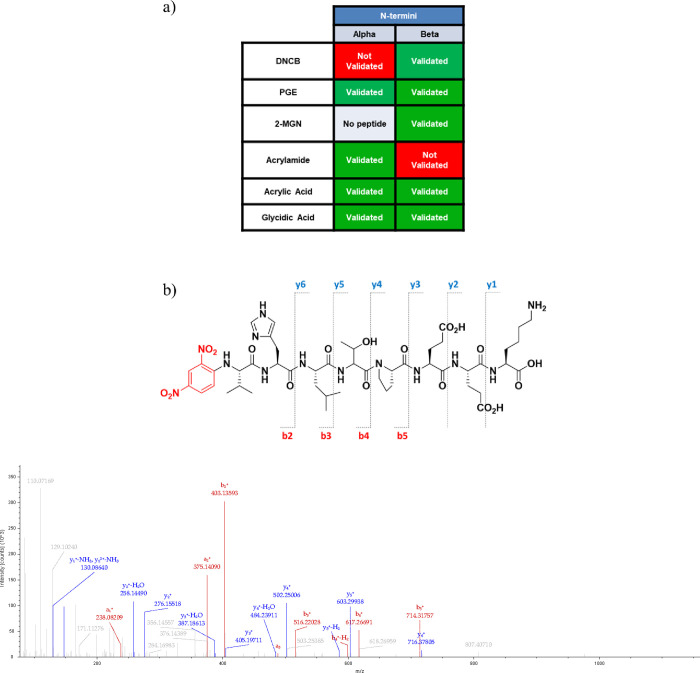
Detection of N-terminal adducts in hemoglobin by bottom-up proteomics. (a) Annotated table demonstrating the formation and localization of N-terminal hemoglobin adducts. Green squares indicate the validated presence of the adduct, red squares indicate the lack of annotated spectra of adducted peptides, and gray squares indicate a lack of detection of the peptide by Proteome Discoverer. (b) Tandem spectrum of beta N-terminal peptide VHLTPEEK showing DNCB adduct formation. B- and y-ions are indicated through red and blue labels, respectively.

In order to determine the utility of bottom-up proteomics mass spectrometry in ascertaining the levels of electrophile adduct formation in hemoglobin, the hemoglobin samples exposed to electrophiles were also processed via tryptic digestion and nanoLC-NSI-MS/MS. Bottom-up proteomics was able to detect the N-terminal adduct formation of all six electrophiles in hemoglobin (Figure 3a). The formation of DNCB adducts at the beta chain N-terminus was confirmed through manual annotation and validation of the MS/MS spectra (see the example in Figure 3b).

**Figure 4:**
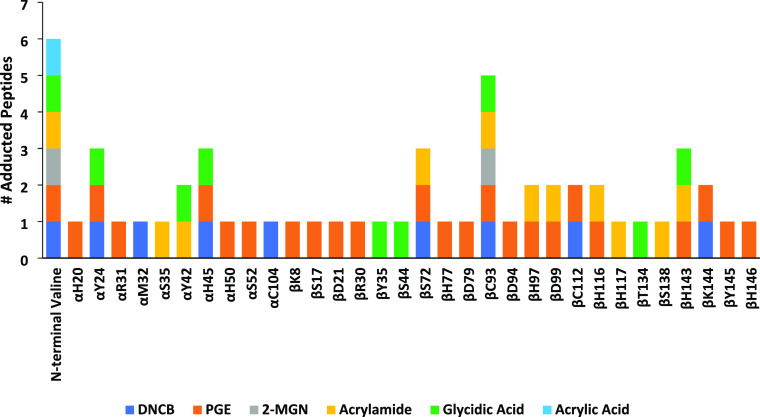
Bottom-up proteomics detect Hb adduct formation at multiple nucleophilic sites. Electrophiles were included as variable modifications in Proteome Discoverer and were manually annotated and validated upon detection by the software and subsequently validated via targeted proteomics.

In addition to the ability of bottom-up proteomics to detect N-terminal hemoglobin adducts, bottom-up proteomics also presents the potential for detection of adducts at additional nucleophilic side chains, a phenomenon that we previously had demonstrated occurred with quinone methide-derived adducts in an earlier study.^8^ The findings are also in good agreement with a previous study conducted by our group where the most reactive sites of hemoglobin with the contact allergens DNCB and PGE were investigated.^28^ In this study, we were able to demonstrate the formation of adducts at multiple nucleophilic side chains, with the Cys93 residue on the beta chain representing the most reactive side chain with five adducts (Figure 4).

Targeted bottom-up proteomics experiments were also conducted to validate the originally detected Hb-electrophile adducts as well as to determine the ability of bottom-up proteomics to quantitate relative levels of adducts without the addition of isotopically labeled internal standards. It was observed that while many of the peptides had a positive correlation between the concentration–time and the amount of adduct detected, many of the adducted peptides did not show a linear relationship, indicating the necessity for authentic isotopically labeled internal standards for accurate quantitation (data not shown).

### Limited Proteolysis Mass Spectrometry Can Detect Coordination Complexes as Well as Covalent Adducts

**Figure 5:**
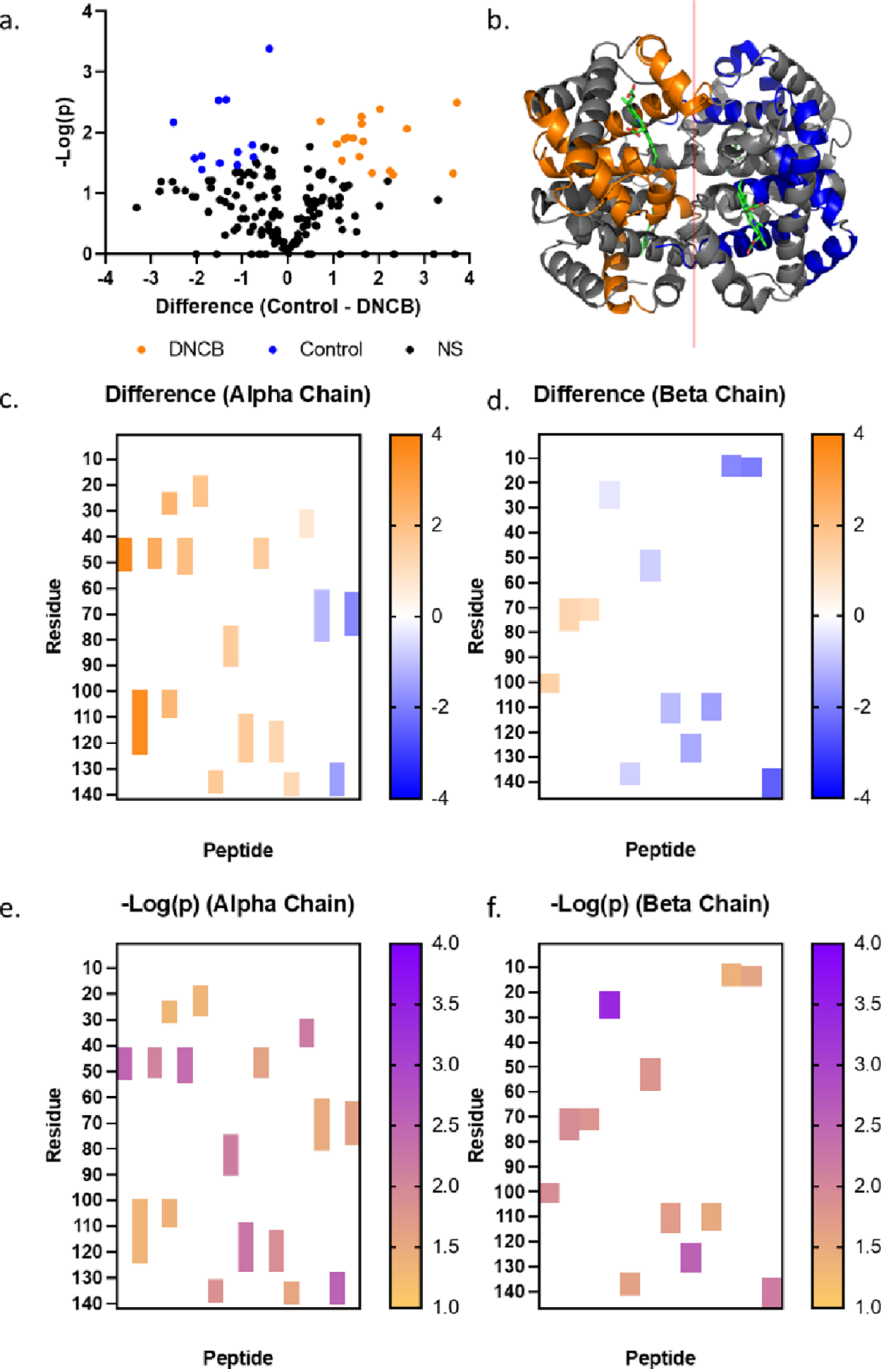
LiP-MS results of the DNCB treatment. (a) Volcano plot of Hb peptide LFQ values of identified following Hb (7.8 μM) treatment with DNCB (39 μM) for 4 h in PBS buffer (pH 7.4) and iterative digestion with proteinase K and then a mixture of trypsin and LysC. Peptides highlighted in orange or blue were significantly enriched in the DNCB or DMSO treatment, respectively, using a statistical cutoff of a 0.05 FDR and a 0.1 minimal coefficient of variation (S0). Data are representative of 3 independent replicates from each treatment condition. (b) Crystal structure of Hb (PBD: 1A3N) with significantly enriched LiP peptides for DNCB or DMSO treatment shown in orange or blue, respectively. (c, d) Heat map of fold change difference of significantly enriched Hb peptides for the alpha or beta chain, respectively. (e, f) Heat map of -log(*p*) of significantly enriched Hb peptides for the alpha or beta chain, respectively.

**Figure 6:**
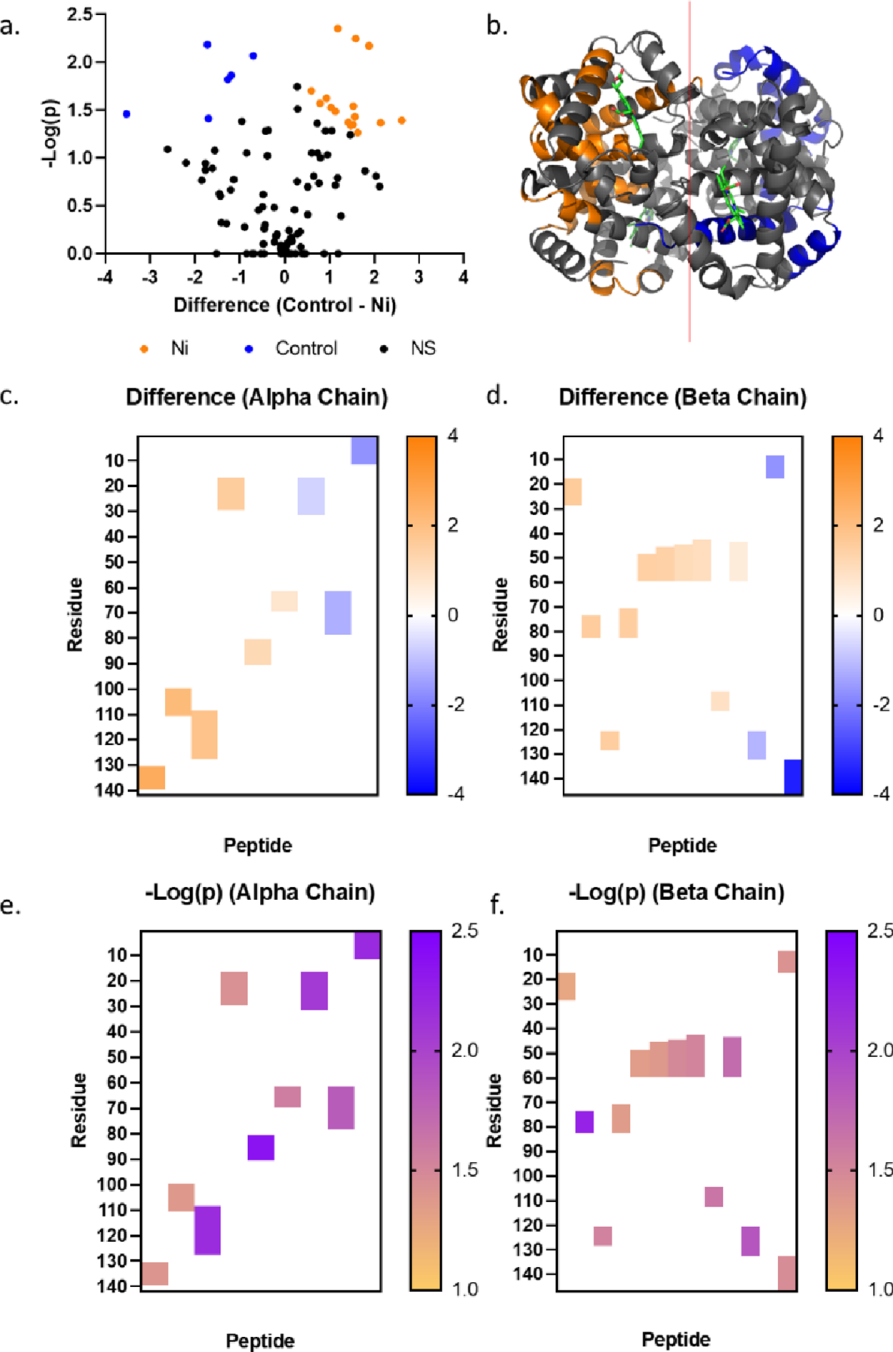
LiP-MS results of the NiCl_2_ treatment. (a) Volcano plot of Hb peptide LFQ values of identified following Hb (7.8 μM) treatment with NiCl_2_ (39 μM) for 4 h in PBS buffer (pH 7.4) and iterative digestion with proteinase K and then a mixture of trypsin and LysC. Peptides highlighted in orange or blue were significantly enriched in the NiCl_2_ or DMSO treatment, respectively, using a statistical cutoff of a 0.05 FDR and a 0.1 minimal coefficient of variation (S0). Data are representative of 3 independent replicates from each treatment condition. (b) Crystal structure of Hb (PBD: 1A3N) with significantly enriched LiP peptides for NiCl_2_ or DMSO treatment shown in orange or blue, respectively. (c, d) Heat map of fold change difference of significantly enriched Hb peptides for the alpha or beta chain, respectively. (e, f) Heat map of -log(p) of significantly enriched Hb peptides for the alpha or beta chain, respectively.

LiP-MS was conducted on hemoglobin exposed to DNCB, PGE, and 2-MGN to compare the ability of this technique to detect adduct formation relative to FI*R*E and conventional bottom-up proteomics (Figure 5). The abundance of limited proteolysis peptides following DNCB exposure was compared to those following DMSO exposure. Seventeen peptides were increased in abundance following DNCB exposure, while 12 peptides show a decrease in abundance (Figure 5a); this corresponds to peptides around the perimeter of the molecule (Figure 5b) throughout the alpha and beta chains (Figures 5c,d). In considering only those regions of the alpha chain that have significant levels of alteration (Figure 6e), we see that this corresponds to regions of the alpha chain between the 20th through 30th residues, as well as the 40th through 50th, 80th through 90th, 110th through 130th, and 130th through 140th. In considering the side chains of the α helix which had validated DNCB adduct formation (Figure 4), we see that this corresponds closely with the residues of Tyr24, His45, and Cys104 seen in the previous experiment. In applying the same scrutiny to the beta chain, we see that the protein regions that are significantly altered (10–30, 50–60, 70–80, 100–105, and 110–150) also encapsulate the Ser73, Cys112, and Lys144 residues observed to have DNCB adducts form in bottom-up proteomics experiments. No significant difference could be shown for hemoglobin treated with either PGE or 2-MGN when the abundance of limited proteolysis peptides was compared with the DMSO-treated control sample (data not shown).

In contrast to DNCB, PGE and 2-MGN, NiCl_2_ is only able to form coordination bonds with the side chains of hemoglobin; however, these interactions have the potential to alter the structure of hemoglobin in a way that limited proteolysis mass spectrometry can discern. Limited proteolysis mass spectrometry of NiCl_2_-treated hemoglobin did show 16 peptides with increased levels and six peptides with decreased levels relative to the control, that is DMSO-treated hemoglobin (Figure 6a); these correspond to a comparable but distinct set of structures on the exterior of the molecule (Figure 6b) found throughout both alpha and beta chains (Figure 6c,d). While there were many sections of both chains that altered their abundance with both DNCB and NiCl_2_ exposure, NiCl_2_ exposure results in a distinct region of increased abundance between residues 50 and 60 on the beta chain (Figure 6f). The sequence of the beta chain in this region contains four lysine residues and a histidine residue, side chains that would theoretically coordinate with the Ni^2+^ ion, thus explaining this site as an adduct formation site.

## Discussion

The high abundance and long half-life of erythrocytes in human blood make hemoglobin an ideal reservoir for the study of exposure-derived protein adducts in human populations. While mass spectrometry technology presents a reliable method for interrogating the hemoglobin adductome, there exist multiple strategies for sample preparation and data analysis of these adducts. In this paper, we set out to examine the relative strengths and weaknesses of the FI*R*E method, untargeted bottom-up proteomics, and limited proteolysis mass spectrometry in characterizing adducts formed from exposing blood to a panel of electrophiles and a metal salt.

The electrophiles used in the present study were chosen due to their history of being detected in human tissues as well as their relevance to human health. Acrylamide is a small electrophilic compound classified as a probable carcinogen by the International Agency for Research on Cancer since 1994^29^ and is found in industrial environments and in food rich in carbohydrates^30^ that has been subjected to the Maillard reaction.^31^ Many studies have demonstrated the presence of acrylamide adducts in human subjects.^11^ Acrylic acid is used in several industrial applications and can also be produced by the Maillard reaction;^32^ it has also been seen to form adducts in hemoglobin.^33^ While glycidic acid is not known to be an environmental contaminant, it is believed to be a precursor of a hydroxypropanoic acid adduct observed in human hemoglobin samples.^34^

The other electrophiles used in this study were chosen due to their documented status as contact allergens, which can induce allergic contact dermatitis (ACD) with repeated exposure. These contact allergens can act as haptens and trigger an immune response when they combine with a carrier protein which the immune system recognizes as “altered self”.^35^ Three of the haptens chosen for this study, DNCB, PGE, and 2-MGN, form covalent adducts with nucleophilic moieties of skin proteins. DNCB is one of the most well-known contact allergens, first recognized as an occupational contact allergen in 1920.^36^ Although not a common reagent, DNCB can be used medically in the treatment of skin growths^37^ and as an intermediate for some industrial products.^38^ PGE is a reactive diluent and a simpler analogue of diglycidyl ether of bisphenol A (DGEBA), which is the most commonly used component of epoxy resins, a class of thermosetting products used as adhesives and coatings and one of the most common causes of occupational ACD.^39,40^ 2-MGN is the biotransformation product of 2-bromo-2-(bromomethyl)-glutaronitrile (MDBGN), a compound introduced in the 1980s as a preservative in industrial and cosmetic products. MDBGN was banned in the EU in 2013 though exposure can still occur via occupational exposure and use of topical medications.^41^ In contrast to the other contact allergens chosen, NiCl_2_ does not form stable covalent bonds with proteins; instead, it forms metal–protein complexes with nucleophilic side chains such as cysteine and histidine, thereby altering the tertiary protein structure. The high prevalence of nickel in jewelry, watches, and glasses makes Ni^2+^ one of the most common contact allergens. Although there is no evidence of hapten-hemoglobin adducts being immunogenic and able to trigger an immune reaction, there are a number of studies that have shown that hapten-hemoglobin adducts can be used as biomarkers to correlate exposure to the hapten with skin and respiratory irritancy/allergy.^42−46^

The analysis of incubated blood samples with FI*R*E showed that four electrophiles – acrylamide, acrylic acid, glycidic acid, and PGE – formed N-terminal adducts with strong dose–responses (Figure 2), indicating its utility for quantitative assessment of the hemoglobin adductome using one or more isotopically labeled internal standards (in this case, AA-Val(D_7_)-FTH). However, FI*R*E was inefficient indetecting N-terminal Val adducts with 2-MGN and failed to detect adducts with DNCB. The decreased ability of FI*R*E to detect these adducts in hemoglobin is likely due to the reaction mechanism that occurs during the FI*R*E procedure, in which the electron pair on the N-terminal amine group of hemoglobin engages in nucleophilic attack on the isothiocyanate carbon in fluorescein isothiocyanate followed by a cyclization and loss of the remainder of the protein (Figure S2a). The bulky 2-MGN adduct may partly occlude the attack of the electron pair on the bulky fluorescein isothiocyanate molecule, slowing the reaction and resulting in lower apparent levels of adduct formation by the FI*R*E method^47^ (Figure S2b). The aromatic nature of the DNCB adduct would result in the partial delocalization of the electron pair in the N-terminal nitrogen within the adduct, theoretically making the FITC derivatization reaction much less likely to occur (Figure S2c). These considerations make the FI*R*E method a powerful, though not universally applicable technique for hemoglobin adductomics.

In contrast to FI*R*E, bottom-up proteomics was able to detect the presence of each of the expected adducts at the N-termini peptides of hemoglobin in blood treated with acrylamide, acrylic acid, glycidic acid, PGE, 2-MGN, and DNCB. The trypsin cleavage site is removed from the N-terminus by several amino acid residues, and thus adduct formation does not interfere with proteolytic activity. In addition, adducts at several additional nucleophilic side chains of the protein were observed (Figure 4). Therefore, the use of bottom-up proteomics expands the utility of hemoglobin as a reservoir for the exposome by offering additional sites to interrogate beyond the N-termini. However, targeted analysis with isotopically labeled internal standards may be needed to allow for the accurate quantification of Hb adducts by bottom-up proteomics.

Our experiments suggest that if the FI*R*E method can be used, this method is much more quantitative than the use of targeted bottom-up proteomics for low levels of adduct formation, while bottom-up proteomics requires higher levels of adduct formation to demonstrate linearity and quantitation. Future experiments will focus on the use of affinity purification of N-terminal peptides to improve the quantitation of low-abundance adducts via targeted bottom-up proteomics.

We also explored the utility of limited proteolysis mass spectrometry in detecting the presence of adducts in hemoglobin following exposure to electrophiles and a metal salt NiCl_2._ We found that when hemoglobin was incubated with DNCB, many of the side chain adducts observed during the bottom-up proteomics experiments were recapitulated during limited proteolysis mass spectrometry. Crucially, the N-termini and βCys93, adducted sites observed in experiments with other electrophiles, were not observed to have altered abundances in response to DNCB exposure and were not flagged as adducted sites. In the case of the N-termini, the surrounding residues had previously been noted to have relatively high solvent accessibilities,^16^ which would have left these regions of hemoglobin vulnerable to proteinase K cleavage regardless of local adduct formation, making them poor candidates for this methodology. By contrast, βCys93 is buried deep within the protein, making it difficult for proteinase K to access. In addition, no significant difference was observed in peptide abundance for hemoglobin treated with either PGE or 2-MGN when compared with DMSO-treated hemoglobin. Taken together, these observations suggest that for protein adductomics, limited proteolysis is best utilized for a subset of residues ideally situated within the protein, which depends on the overall protein structure. Where limited proteolysis outperforms FI*R*E and bottom-up proteomics, is in the detection of metal adducts, as in the case of hemoglobin exposure to NiCl_2_ where several coordination sites were observed. It should be noted, however, that limited proteolysis mass spectrometry does not determine the *m*/*z* of the hemoglobin adducts, and the electrophiles/metals must therefore be known or identified in advance of analysis, making this method unsuitable for discovery experiments.

Hemoglobin adducts induced by electrophilic compounds can occur through different exposure routes, as ingestion, inhalation, or dermal contact. The GC/MS-based modified Edman procedure has been used to quantify increments in Hb adduct levels of acrylamide and glycidamide after exposure for a few days to normal food with high content of acrylamide.^48^ The method has also been used to measure acrylamide exposure from food in the general population^11^ and to quantify adduct levels of acrylamide (20–100 pmol/g globin) and glycidamide (40–140 pmol/g globin) in nonsmoking adults.^49^ In a study by Hagmar et al., the same type of modified Edman method was used to correlate levels of acrylamide-Hb adducts in blood to irritative skin symptoms after dermal exposure to acrylamide.^42^ The LC/MS based FI*R*E procedure has, e.g., been used to quantify adducts from acrylamide, glycidamide, and ethylene oxide in nonsmoking/smoking mothers and their newborns (adduct levels range from 5 pmol/g Hb in nonsmokers, up to a few hundred in smokers).^50^ Additionally, this method has successfully screened for unknown Hb adducts between smokers and nonsmokers, capable of detecting adducts from 10 pmol/g hemoglobin to 1.2 nmol/g hemoglobin.^51^ Bottom-up proteomics has also been able to detect Hb adducts in smokers through the use of appropriate internal standards.^18^ These studies highlight the versatility of Hb adductomics and inspire the continuous innovation of this approach to enable it to be used routinely in toxicant exposure and biomarker assessment. While our study benchmarks three different MS techniques against each other in analysis of blood samples exposed to electrophiles, we expect these results to translate to real-world exposures. Given the sensitivity of FI*R*E and the versatility of bottom-up proteomics toward Hb adducts, we envision that Hb adductomics may also have further utility in detecting adducts generated through skin contact in future work.

## Conclusions

Our results indicate that FI*R*E, bottom-up proteomics, and LIP-MS provide different but complementary information about hemoglobin adducts in human samples (Table 2). The FI*R*E methodology is accurate and reliable in detecting minute quantities of N-terminal adducts; however, this methodology is limited in its ability to determine the location of adducts on the hemoglobin molecule and fails to detect bulkier aromatic electrophile adducts. By contrast, bottom-up proteomics can detect adducts stemming from a wider variety of electrophiles and determine their locations within the alpha and beta side chains of the protein but does not provide accurate quantification in the absence of an isotopically labeled standard. Both methods can be used with untargeted and targeted experiments and are readily made quantitative with the use of appropriate internal standards, though further research is needed to determine whether specific internal standards are needed for each adduct or whether semiquantitation with homologous standards is sufficiently accurate for untargeted quantitation. Finally, limited proteolysis mass spectrometry can determine the presence of both electrophilic and metal adducts present in hemoglobin, although at present the analysis of the adducts relies on the knowledge of what electrophiles/metals are present; future experiments to couple limited proteolysis mass spectrometry to inductively coupled plasma mass spectrometry (ICP-MS) of the same samples would allow for the identification of coordinating metals in hemoglobin as well as where they are on the molecule. ICP-MS has for instance been used to identify trivalent arsenic coordination binding of hemoglobin cysteine side chains by Lu et al.^52−54^ With this information in hand, it is our position that the method of choice for examining the hemoglobin adductome is largely dependent on the question asked and the desired information. In addition, the methods discussed here are by no means limited to hemoglobin but can also be extended to other blood proteins,^55^ mucus proteins,^56^ etc.

**Table 2:** Summary of the Potential of the Three Methods to Assay Hemoglobin Adducts

	FI*R*E	bottom-up proteomics	limited proteolysis
discovery/identification?	++	+++	–
bulky/electron-withdrawing adducts?	–	+++	++
quantitative?	+++	+	–
covalent adducts?	++	+++	++
coordination complexes?	–	–	+++

## Supporting Information Available

Results from the FI*R*E procedure for 2-MGN and DNCB (Figure S1); suggested reaction mechanisms for 2-MGN and DNCB that occurs during the FI*R*E procedure (Figure S2) (PDF)
