# Supplementary material for: Mass Spectrometry-Based Strategies for Assessing Human Exposure Using Hemoglobin Adductomics
Source: Chem Res Toxicol. 2023 Nov 14;36(12):2019–30. doi: 10.1021/acs.chemrestox.3c00294 (PMC10731639; doi:10.1021/acs.chemrestox.3c00294)
Supplement: Supplementary file 1 — tx3c00294_si_001.pdf [file tx3c00294_si_001.pdf]

Supporting Information

Mass Spectrometry-Based Strategies for Assessing Human Exposure Using Hemoglobin Adductomics

Andrew T. Rajczewski[1#], Lorena Ndreu[2#], Efstathios Vryonidis[2#], Alexander K. Hurben[3], Sara Jamshidi[2], Timothy J. Griffin[1], Margareta Törnqvist[2], Natalia Y. Tretyakova[3*], Isabella Karlsson[2*]

[1]Department of Biochemistry, University of Minnesota, Minneapolis, Minnesota 55455, United States

[2]Department of Environmental Science, Stockholm University, SE-10691 Stockholm, Sweden.

[3]Department of Medicinal Chemistry and the Masonic Cancer Center, University of Minnesota, Minneapolis, Minnesota 55455, United States

[#]*These authors contributed equally.*

***Corresponding Authors**

Natalia Tretyakova, Department of Medicinal Chemistry and the Masonic Cancer Center, University of Minnesota, Minneapolis, Minnesota 55455, United States, email: trety001@umn.edu

Isabella Karlsson, Department of Environmental Science, Stockholm University, SE-10691 Stockholm, Sweden, email: Isabella.Karlsson@aces.su.se

TABLE OF CONTENTS

2-MGN AND DNCB ADDUCTS ARE LESS EFFECTIVELY DETECTED USING THE FI*R*E METHOD 3

Figure S1: Results from the FIRE procedure for 2-MGN and DNCB. 3

Figure S2: Suggested reaction mechanisms for 2-MGN and DNCB that occurs during the FIRE method. 4

2-MGN and DNCB adducts are less effectively detected using the FI*R*E method

a)

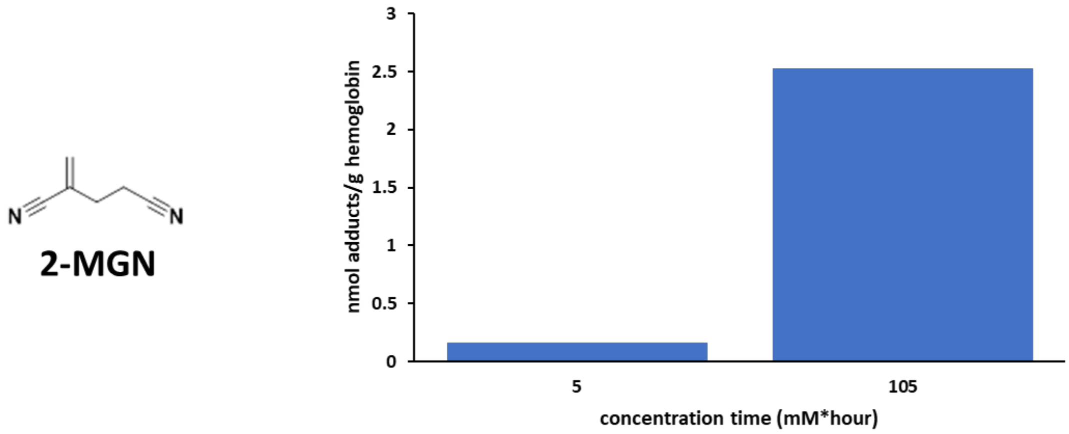

b)

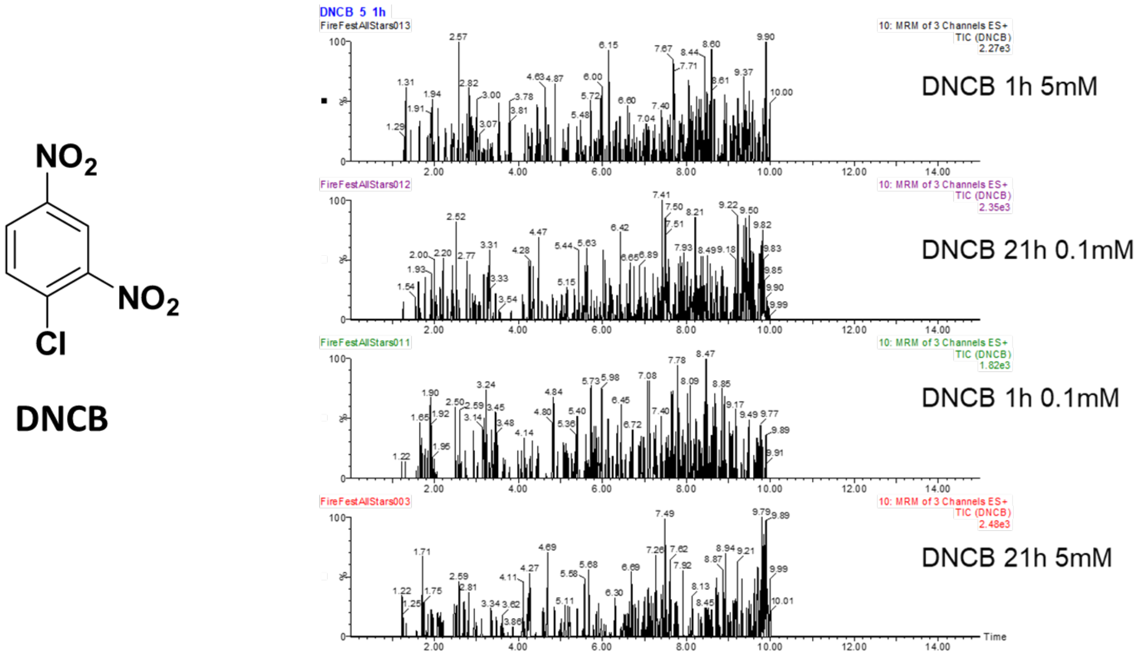

Figure S1: *Results from the FIRE procedure for 2-MGN and DNCB.*
a) Normalized signals for 2-MGN-Val-FTH (related to concentration over time at incubation) b) Total ion chromatograms showing a lack of DNCB-Val-FTH formation.

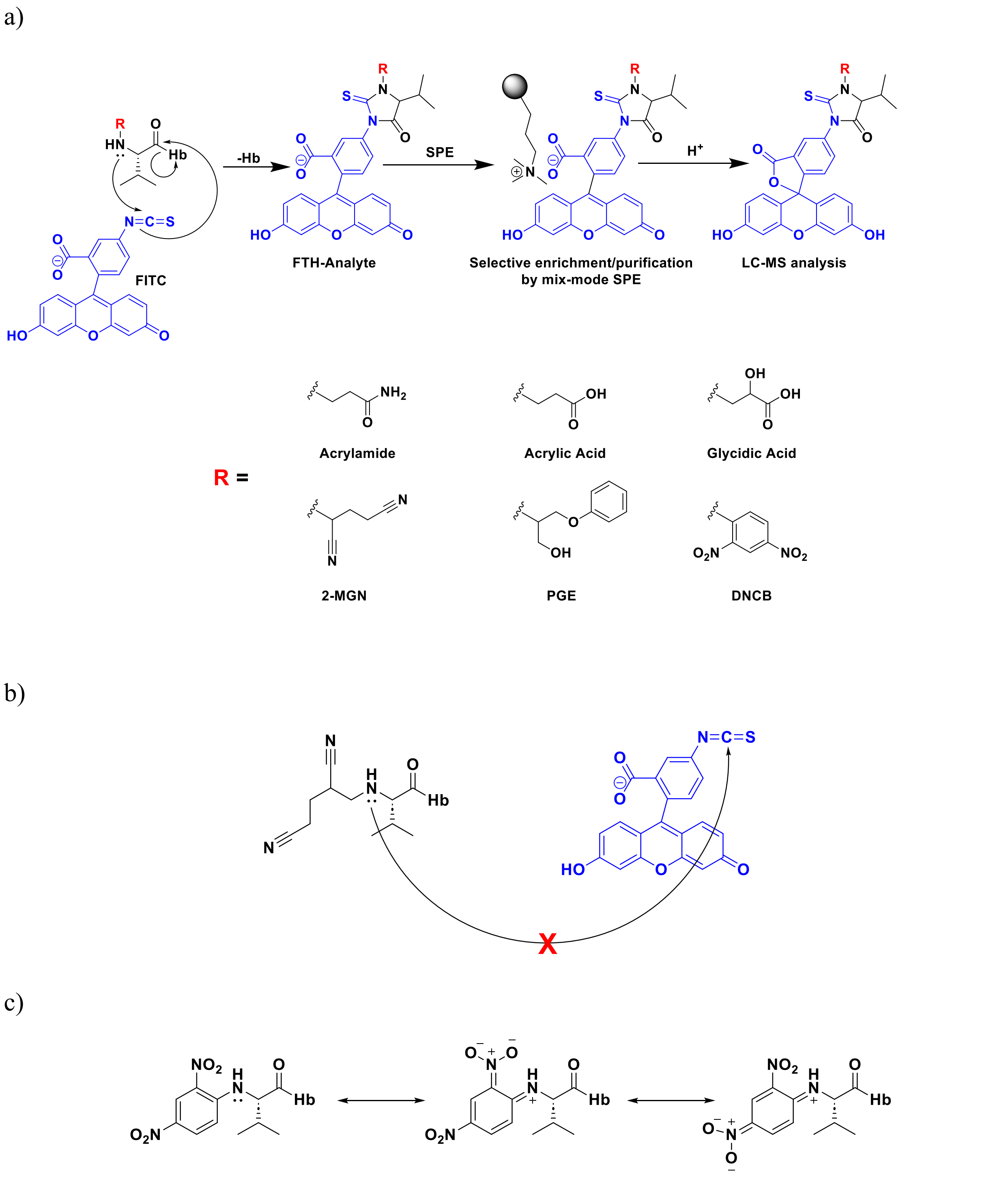

Figure S2: *Suggested reaction mechanisms for 2-MGN and DNCB that occurs during the FIRE method. a) The FIRE method reaction. The structure of the different adducts (R) and the name of the corresponding precursor electrophiles. b) The large size of 2-MGN limits interaction with FITC. c) The delocalized electron pair in the DNCB adduct precludes DNCB-Val-FTH formation.*
